# Functional inactivation of *Plasmodium falciparum* glycogen synthase kinase GSK3 modulates erythrocyte invasion and blocks gametocyte maturation

**DOI:** 10.1016/j.jbc.2022.102360

**Published:** 2022-08-10

**Authors:** Arne Alder, Louisa Wilcke, Emma Pietsch, Heidrun von Thien, Samuel Pazicky, Christian Löw, Paolo Mesen-Ramirez, Anna Bachmann, Paul-Christian Burda, Conrad Kunick, Holger Sondermann, Danny Wilson, Tim-Wolf Gilberger

**Affiliations:** 1 CSSB Centre for Structural Systems Biology, Hamburg, Germany; 2 Department of Cellular Parasitology, Bernhard Nocht Institute for Tropical Medicine, Hamburg, Germany; 3 Department of Biology, University of Hamburg, Hamburg, Germany; 4 Hamburg Unit, European Molecular Biology Laboratory, Hamburg, Germany; 5 Institut für Medizinische und Pharmazeutische Chemie, Technische Universität Braunschweig, Braunschweig, Germany; 6 Deutsches Elektronen-Synchrotron DESY, Germany; 7 Christian-Albrechts-Universität zu Kiel, Kiel, Germany; 8 Research Centre for Infectious Diseases, School of Biological Sciences, University of Adelaide, South Australia, Australia; 9 Burnet Institute, Melbourne, Victoria, Australia; 10 Institute for Photonics and Advanced Sensing (IPAS), University of Adelaide, South Australia, Australia

**Keywords:** malaria, *Plasmodium falciparum*, GSK3, kinase, host cell invasion, gametocytogenesis, AMA1, apical membrane antigen 1, CDPK, calcium-dependent protein kinase, CK2, casein kinase 2, FL, full-length, GDV1, gametocyte development 1, GSK3, glycogen synthase kinase 3, MAP, mitogen-activated protein, PDK1, 3-phosphoinositide-dependent protein kinase 1, PKA, protein kinase A, RBC, red blood cell, SLI, selection-linked integration, TGD, targeted gene disruption

## Abstract

Malaria is responsible for hundreds of thousands of deaths every year. The lack of an effective vaccine and the global spread of multidrug resistant parasites hampers the fight against the disease and underlines the need for new antimalarial drugs. Central to the pathogenesis of malaria is the proliferation of *Plasmodium* parasites within human erythrocytes. Parasites invade erythrocytes *via* a coordinated sequence of receptor–ligand interactions between the parasite and the host cell. Posttranslational modifications such as protein phosphorylation are known to be key regulators in this process and are mediated by protein kinases. For several parasite kinases, including the *Plasmodium falciparum* glycogen synthase kinase 3 (PfGSK3), inhibitors have been shown to block erythrocyte invasion. Here, we provide an assessment of PfGSK3 function by reverse genetics. Using targeted gene disruption, we show the active gene copy, PfGSK3β, is not essential for asexual blood stage proliferation, although it modulates efficient erythrocyte invasion. We found functional inactivation leads to a 69% decreased growth rate and confirmed this growth defect by rescue experiments with wildtype and catalytically inactive mutants. Functional knockout of PfGSK3β does not lead to transcriptional upregulation of the second copy of PfGSK3. We further analyze expression, localization, and function of PfGSK3β during gametocytogenesis using a parasite line allowing conditional induction of sexual commitment. We demonstrate PfGSK3β-deficient gametocytes show a strikingly malformed morphology leading to the death of parasites in later stages of gametocyte development. Taken together, these findings are important for our understanding and the development of PfGSK3 as an antimalarial target.

Malaria is one of the most dangerous infectious diseases worldwide with more than 241 million cases and 627,000 deaths in 2020 (1). The disease is caused by protozoan parasites of the genus *Plasmodium* that are transmitted to humans by female *Anopheles* mosquitoes. The malaria parasite possesses a complex life cycle that alternates between sexual reproduction in the mosquito vector and asexual replication within the vertebrate host. During infection, the proliferation of asexual stages in human erythrocytes is responsible for all clinical symptoms of the disease. Control measures like drug treatment and prophylaxis, improved rapid diagnostic tests as well as vector control strategies have led to great success in the reduction of worldwide malaria cases since 2000 (1, 2). Unfortunately, this trend has stalled since 2015 (1, 2). Due to the limitations in currently available vaccine efficacy, malaria control and treatment in humans is still reliant on access to antimalarial drugs. Artemisinin-based combination therapy is recommended by the WHO as the first line treatment for severe malaria cases. However, resistance to artemisinin is spreading, and alarmingly, there is increasing resistance to licensed artemisinin-based combination therapies (3). Consequently, new antimalarial chemotypes with novel modes of action are urgently needed.

During the disease causing blood stage of malaria, the parasite invasion of red blood cells (RBCs) represents an attractive intervention point for antimalarial drugs (4). Protein kinases possess a strong potential as antimalarial targets given their crucial role for parasite survival (5). A substantial body of work highlights the role of this enzyme class in the regulation of host cell invasion (6, 7, 8, 9, 10, 11, 12, 13, 14, 15, 16). For instance, the physiological role of protein kinase A, casein kinase 2 (CK2) as well as glycogen synthase kinase 3 (GSK3) during the host cell invasion has been demonstrated (6, 7, 8, 9, 10, 16, 17, 18, 19). These invasion-related kinases target the cytoplasmic domains of so-called invasins (6, 8, 9, 10, 16)—proteins that are secreted during the invasion process and mediate binding to surface receptors on the host cell.

GSK3 is a serine/threonine kinase that is ubiquitously present in Metazoa. The human genome encodes two different isoforms of the kinase, which are both essential and involved in a variety of cellular processes such as glycogen metabolism, regulation of the cell cycle, translational regulation, embryonic development, or differentiation of neurons (20, 21). The genome of *P. falciparum* encodes two orthologs (PF3D7_0312400 and PF3D7_1316000). PF3D7_0312400 (PfGSK3β) shows higher sequence similarity to the beta-isoform of human GSK3. PF3D7_1316000 (PfGSK3α) also has similarity to the GSK3-family (22) and therefore represents a potential second isoform of the kinase. However, only PF3D7_0312400 is expressed in asexual blood stages (23, 24, 25, 26).

In previous studies, PfGSK3β was considered as likely essential in asexual stages of the parasite and investigated as a potential drug target for the development of antimalarial kinase inhibitors (10, 27, 28, 29, 30, 31, 32, 33, 34).

Prior to RBC invasion, it was reported that PfGSK3β phosphorylates the cytoplasmic domain of apical membrane antigen 1 (PfAMA1), a microneme localized secreted protein that mediates the essential step of “tight junction” formation with the host cell membrane (7, 8, 9, 10, 16, 35). PfGSK3-dependent phosphorylation of PfAMA1 requires a prephosphorylation event by protein kinase A (PfPKA) (7, 8, 9, 10, 16) and its chemical inhibition leads to reduced RBC invasion (8, 10, 36).

Recent studies provided evidence that protein kinases play an important role in gametocytogenesis and the subsequent formation of gametes (17, 19, 37, 38). Gametocytes are sexually committed parasite stages that are able to leave the intraerythrocytic proliferation cycle and are transmissible to the mosquito vector (39, 40). This development requires about 10 days and is accompanied not only by distinct morphological changes but also by an extensive reprogramming of the parasite’s cellular metabolism. The importance of kinases during the process provides additional potential drug targets for the development of transmission-blocking chemotypes (41).

Here we report on the detailed functional characterization of PfGSK3β in its cellular context during asexual multiplication and gametocytogenesis.

## Results

### PfGSK3β-deficient parasites are viable

**Figure 1:**
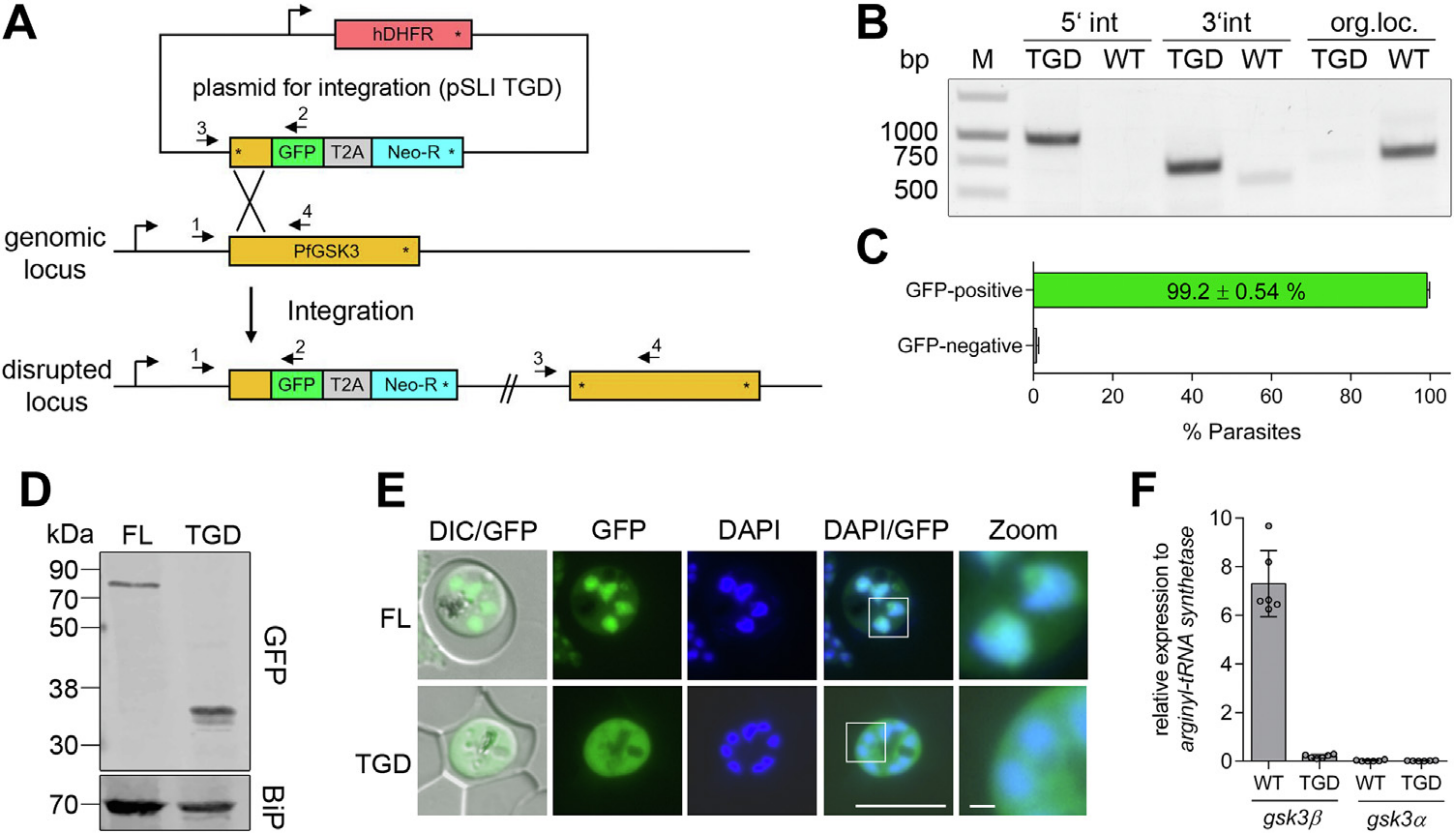
**PfGSK3β**-**deficient parasites are viable**. *A*, schematic representation of selection-linked integration (SLI) strategy used to generate a functional knock out of PfGSK3β. Binding sites of primers used to confirm correct genomic integration are indicated by *black arrows*. TGD, targeted gene disruption; hDHFR, human dihydrofolate reductase; GFP, green fluorescent protein; T2A, skip peptide; Neo-R, neomycin-resistance gene; *angled arrows* = promoters; *asterisks* = stop codon. *B*, integration PCR with genomic DNA isolated from PfGSK3^TGD^ (TGD) or parental 3D7 (WT) parasites. Primer combinations: 5′int (1 + 2); 3′int (3 + 4); original locus (1 + 4). Position of primers as indicated in (*A*). Expected PCR products: 1 + 2 = 969 bp; 3 + 4 = 713 bp; 1 + 4 = 743 bp. *C*, quantification of GFP-positive parasites in PfGSK3β^TGD^. Bar graph represents mean ± SD of five independent occasions. In total, 1409 infected erythrocytes were analyzed. *D*, detection of full-length (FL, ∼80 kDa) and disrupted (TGD, ∼38 kDa) PfGSK3β by immunoblotting using anti-GFP antibodies. BiP (70 kDa) was used as a loading control. *E,* localization of endogenously GFP-tagged full-length (PfGSK3β^FL^) and disrupted (PfGSK3β^TGD^) kinase was analyzed by fluorescence microscopy in unfixed parasites. Nuclei were stained with DAPI. *White squares* refer to magnified sections presented in the rightmost images. DIC, differential interference contrast. Scale bar = 8 μm (zoom 1 μm). *F*, expression of full-length PfGSK3 isoforms in 3D7 wildtype (WT) *versus* PfGSK3β^TGD^ schizonts (40 hpi) analyzed by qPCR. Expression was calculated relative to *arginyl-tRNA synthetase*. Bar graphs represent mean ± SD of triplicates from two independent experiments.

**Figure 2:**
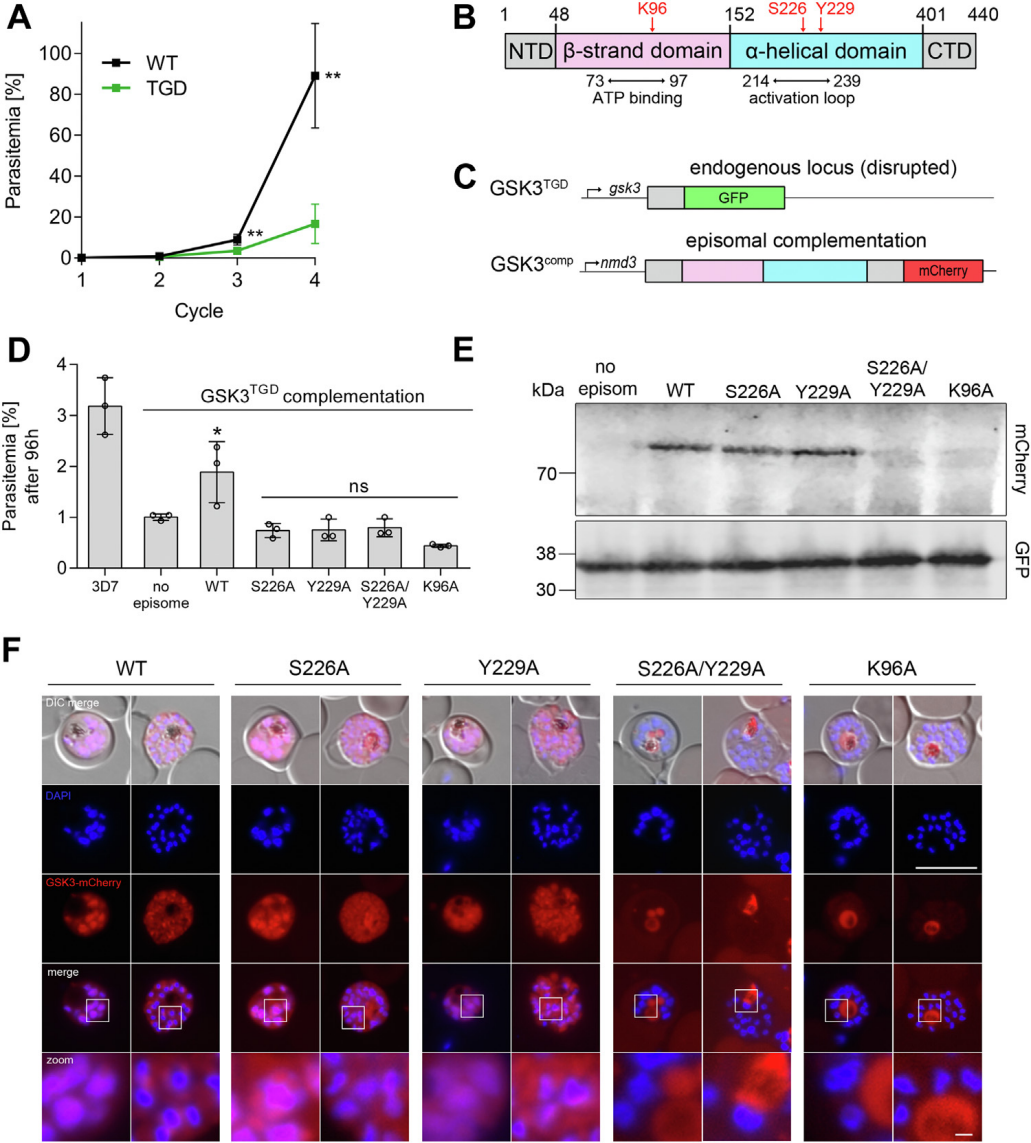
**PfGSK3β catalytic activity is important for efficient parasite proliferation**. *A*, growth analysis of synchronous PfGSK3β^TGD^ and 3D7 wildtype parasites (WT) over four replication cycles measured by flow cytometry. In cycle 3, parasites were diluted to prevent overgrowth of the culture. Parasitemia was calculated as cumulated values. Data points represent mean ± SD from five independent experiments. Significance was analyzed in GraphPad Prism using unpaired *t* test. Statistically significant differences are indicated (∗∗*p* < 0.01). *B*, schematic representation of PfGSK3β domains. B-strand domain and α-helical domain of the kinase are indicated in *pink* and *blue*, respectively. Essential residues in the ATP-binding region (K96) and phosphorylated residues of the activation loop (S226, Y229) are indicated in *red*. Amino acid numbers as indicated. NTD, N-terminal domain; CTD, C-terminal domain. *C*, complementation of PfGSK3β^TGD^ with episomally expressed full-length PfGSK3β-mCherry under the constitutive *nmd3* promoter. *D*, growth analysis of 3D7 wildtype parasites (3D7) and PfGSK3β^TGD^ (no episome) in comparison to complemented PfGSK3β^TGD^. WT, episomal complementation with the canonical version of PfGSK3β. Additionally, PfGSK3β^TGD^ was complemented with different episomally expressed mutants (S226A; Y229A; S226A/Y229A; K96A). Parasitemia of synchronous parasite cultures after 96 h (starting parasitemia 0.1%) was measured by flow cytometry. Bar graphs represent mean ±SD of three independent experiments. Significance of complemented parasites compared to “no episome” was analyzed by one-way ANOVA and Dunnett’s multiple comparison test ∗*p* < 0.05; ns, not significant. *E*, episomally expressed versions of PfGSK3β-mCherry (∼80 kDa, point mutations as indicated) were detected using anti-RFP antibodies. PfGSK3β^TGD^ without episomal complementation (no episome) was used as a negative control. Endogenous PfGSK3β^TGD^-GFP (∼38 kDa) was detected using anti-GFP antibodies. *F*, localization of episomally expressed canonical PfGSK3β-mCherry and PfGSK3β mutants (point mutations as indicated) analyzed by fluorescence microscopy. Nuclei were stained with DAPI. *White frames* refer to magnified sections presented at the bottom of each panel. DIC, differential interference contrast. Scale bar = 8 μm (zoom 1 μm). TGD, targeted gene disruption.

Reverse genetic approaches to probe into the essentiality of GSK3 in the malaria parasite from previous knockout and mutagenesis screens report inconsistent findings (27, 28, 42, 43, 44). Therefore, we aimed to revalidate the potential physiological redundancy of PfGSK3β in *P. falciparum* by targeted gene disruption (TGD) (45). To do so, we used a 572 bp 5′ region including a 335 bp intronic region of *pfgsk3β* to enable homologous recombination and integration of the targeting plasmid into the endogenous genomic locus. (Fig. 1*A*). This recombination results in the expression of an inactive version of the kinase devoid of essential domains including the ATP-binding site (K96), the catalytic site (D194), and activating phosphorylation motifs (Y229) but with a C-terminal–fused GFP (Figs. 1*A* and 2*C*). We show that upon selection with G418, that selects for plasmid integration and gene disruption, we were able to obtain viable parasites. The cell line was subsequently subjected to limiting dilution cloning, and experiments were performed with cloned parasites. First, we confirmed the *pfgsk3β* locus was disrupted by PCR (parasite line referred to as PfGSK3β^TGD^, Fig. 1*B*). To quantify revertant WT parasites in this clonal population, we analyzed the proportion of GFP-positive parasites by fluorescence microscopy (Figs. 1*C* and S2). Our analysis showed that 99.2 ± 0.54% of the parasites are GFP-positive (Fig. 1*C*) confirming that vast majority of PfGSK3β^TGD^ do have the pSLI vector integrated leading to the expression of GFP (Fig. S2). Using the same selection linked integration system (45), we generated a second cell line with full-length (FL) PfGSK3β endogenously GFP-tagged at the C terminus (referred to as PfGSK3β^FL^, Fig. S1*A*) as a control and confirmed integration by PCR (Fig. S1*B*).

Expression of the truncated or tagged kinase (PfGSK3β^TGD^ *versus* PfGSK3β^FL^) was confirmed by Western blotting. As expected, the GFP-tagged PfGSK3β^FL^ migrates in an SDS-PAGE gel at 80 kDa (calculated MW of 80.2 kDa) and truncated PfGSK3β^TGD^ was detected at 35 kDa (calculated MW 37.9 kDa) (Fig. 1*D*).

PfGSKβ^FL^ and PfGSK3β^TGD^ localization was analyzed by fluorescence-based imaging. As previously reported for PfGSK3β (10), PfGSK3β^FL^ predominantly localizes to the nucleus of trophozoites and the nuclear periphery of schizonts with some PfGSK3β^FL^ also evident in the cytosol. However, upon disruption of PfGSK3, the truncated PfGSK3β^TGD^ version of the kinase appears solely cytosolic (Figs. 1*E*, 2*F* and S2*B*).

To assess a putative functional compensation of PfGSK3β by an upregulation of PfGSK3α (PF3D7_1316000), we performed qPCR analysis (Fig. 1*F*). We show that disruption of PfGSK3β does not influence the expression profile of the second α-isoform as neither PfGSK3β^TGD^ nor wildtype (WT) parasites showed any expression of PfGSK3α detectable by qPCR (Fig. 1*F*).

Taken together, our data highlight that PfGSK3β is not essential for asexual parasite proliferation and its first 80 amino acids are not sufficient to mediate a PfGSK3β-specific localization in the parasite.

### PfGSK3β catalytic activity is important for efficient parasite proliferation

Next, we analyzed the proliferation of PfGSK3β-deficient parasites over four replication cycles and compared it with a 3D7 WT control. Parasite proliferation was quantified by flow cytometry revealing a decreased proliferation rate. After four replication cycles, PfGSK3β^TGD^ parasites showed an 81.3 ± 9.4% reduction of growth compared to the control parasites implying an important although not essential role of the kinase for efficient parasite proliferation *in vitro* (Fig. 2*A*).

To verify that this reduction in parasite multiplication represents the phenotypic consequences of the functional inactivation of PfGSK3β, we complemented PfGSK3β^TGD^ with an episomal copy expressed as mCherry-fusion under the constitutive *nmd3* promoter (45) (Fig. 2*C*). As expected, in complemented PfGSK3β^TGD^ parasites, the episomally expressed PfGSK3β^WT^-mCherry localized predominantly in the nuclear periphery of trophozoites and schizonts (Fig. 2*F*). Notably, in WT-complemented parasites, the observed multiplication defect was reversed, and parasite proliferation was partially restored (68.5 ± 1.6% growth reduction in PfGSK3β^TGD^ *versus* 41.7 ± 15.4% growth reduction in complemented parasites, Fig. 2*D*), supporting the specificity of PfGSK3β deletion phenotype (Fig. 2*A*).

In addition, we applied our complementation approach to elucidate the physiological consequences of PfGSK3β point mutants. As recently described with recombinant PfGSK3β, a conserved residue in the ATP-binding region (K96) as well as two phosphorylation sites (S226, Y229) in the activation loop of the enzyme (Fig. 2*B*) are essential for catalytic activity and integrity/solubility of the kinase (46). Expression of PfGSK3β featuring single point mutants (K96A, S226A, Y229A) or an activation loop-double mutant (S226A/Y229A) could not restore the growth defect in PfGSK3β^TGD^ parasites (Fig. 2*D*). To further analyze this phenotype, we probed into the expression of these mutants by Western blotting and fluorescence microscopy (Figs. 2, *E* and *F* and S3). Only the S226A-GSK3β and Y229A-GSK3β variants were detectable by Western blotting as FL proteins (calculated MW 80 kDa), with a faint additional band at 26 kDa (most likely corresponding to cleaved mCherry, Fig. S3) visible using anti-RFP antibodies. In contrast, no signal was detected for K96A and S226/Y229A indicating that mutation of these residues impairs stability of the kinase (Figs. 2*E* and S3). In line with this finding, only S226A-GSK3β and Y229A-GSK3β variants showed a localization in the parasite resembling that of WT PfGSK3β (Fig. 2*F*). Consistent with our Western blot analysis, in K96A- and S226A/Y229A-complemented parasites, no fluorescence signal was detectable in the cytosol. Some signal is visible in close proximity to the hemozoin crystal in the parasite, and this could be explained by autofluorescence of the food vacuole (Fig. 2*F*).

### Erythrocyte invasion is modulated by PfGSK3β activity

**Figure 3:**
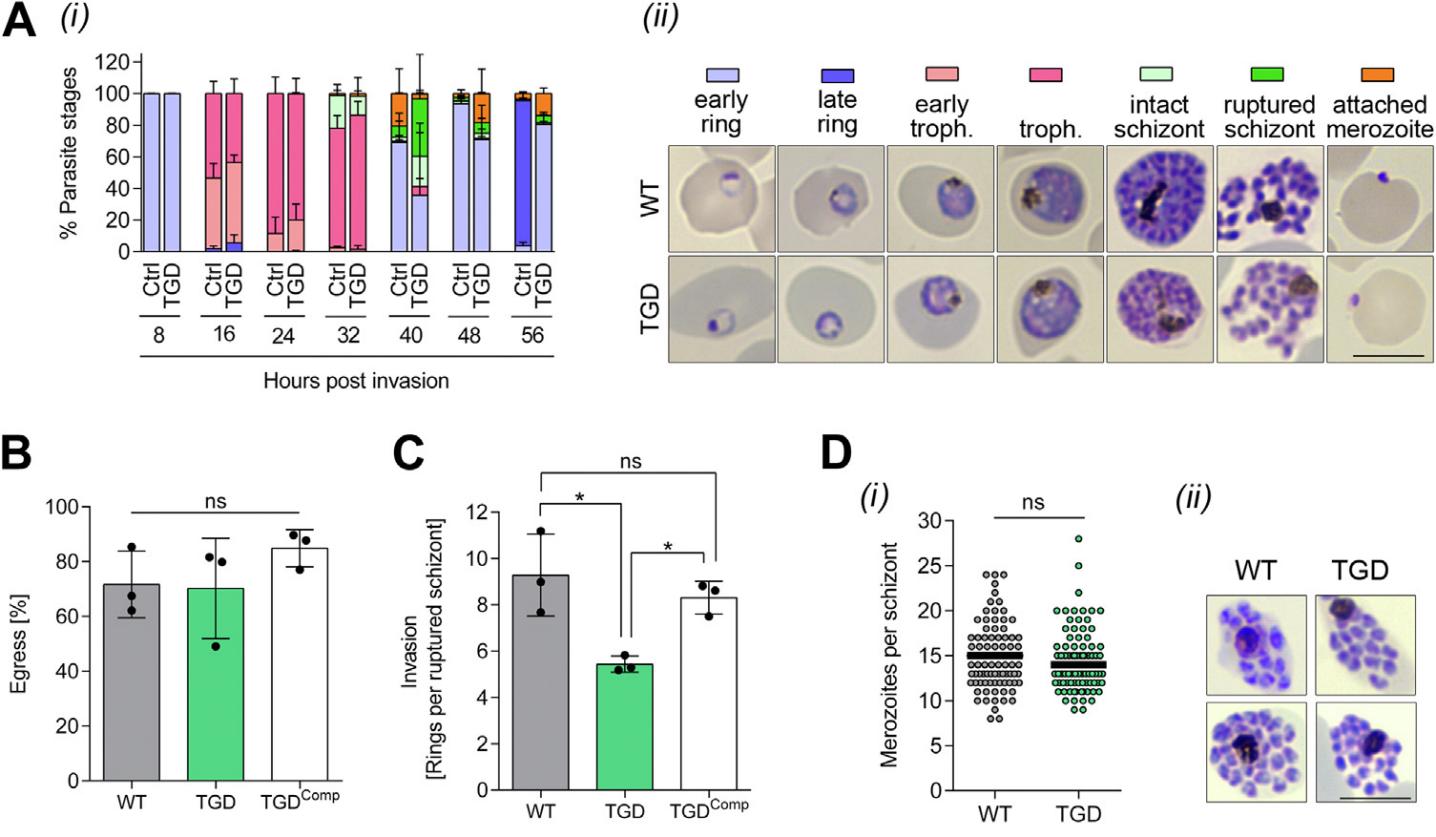
**Erythrocyte invasion is modulated by PfGSK3β activity**. *A*, stage transition of PfGSK3β^TGD^ and 3D7 wildtype (WT) parasites. (i) Parasite stages were counted every 8 h over one replication cycle in Giemsa-stained blood smears. Percentages represent mean ± SD from three independent experiments. For all time points, a total number of at least 1000 parasites were counted. (ii) Representative images of Giemsa-stained blood smears as quantified in (i). Scale bar = 8 μm. *B* and *C*, egress and invasion of PfGSK3β^TGD^, 3D7 wildtype parasites (WT) and WT-complemented PfGSK3β^TGD^ (TGD^Comp^) were analyzed in highly synchronous cultures. Schizont and ring parasitemia were quantified by counting parasites in Giemsa-stained blood smears at 44 hpi and 52 hpi. Egress was quantified as reduction of schizont parasitemia between both time points. Invasion was calculated as newly formed rings per ruptured schizont. All data points represent mean ± SD of three independent experiments. For each experiment at least 5000 cells were quantified. Significance was analyzed in GraphPad Prism using one-way ANOVA and Tukey's multiple comparisons test. Statistically significant differences are indicated (∗*p* < 0.05; ns, not significant). *D*, merozoites per schizont were counted in Giemsa-stained blood smears of segmented schizonts from PfGSK3β^TGD^ and 3D7 wildtype cultures. (i) In total, 96 schizonts from three independent experiments were analyzed for both PfGSK3β^TGD^ and WT control. *Black lines* represent means. Significance was analyzed in GraphPad Prism using unpaired two-tailed *t* test. (ii) Representative images of segmented schizonts in Giemsa-stained blood smears as quantified in (i). Scale bar = 8 μm. TGD, targeted gene disruption.

To further dissect the growth phenotype of the PfGSK3β-deficient parasites and to assess the physiological function of PfGSK3β, we investigated parasite maturation and stage conversion. First, parasites were analyzed every 8 h during one replication cycle in Giemsa-stained blood smears of a highly synchronous culture. PfGSK3β^TGD^ parasites showed normal transition from ring stages to trophozoites and schizonts during intraerythrocytic development with a modest delay in comparison to control parasites (Fig. 3*A*). However, the slightly slower development of PfGSK3β^TGD^ parasites could not account for the observed defect in parasite multiplication.

Second, since PfGSK3β was shown to be involved in invasion *via* its phosphorylation of the essential invasion ligand AMA1, we quantified invasion (and egress) of PfGSK3β^TGD^ and compared it with WT control parasites. For this, we counted schizonts and ring stage parasites in Giemsa-stained blood smears of highly synchronous pre-egress and postreinvasion cultures. As shown in Figure 3, *B* and *C*, only host cell invasion was significantly reduced in PfGSK3β^TGD^ (42.9 ± 3.01% reduced invasion compared to the control). In contrast, when PfGSK3β^TGD^ parasites were complemented with an episomal WT-copy of PfGSK3β (Fig. 2*C*), efficient erythrocyte invasion was partially restored (11.8 ± 6.02% reduced invasion compared to the control, Fig. 3*C*). This is in line with our initial complementation assay measuring parasite growth (Fig. 2*D*) and further links the observed defect in erythrocyte invasion to the functional inactivation of PfGSK3β.

Since the human ortholog of GSK3β was shown to be involved in controlling cell cycle progression (21), we next quantified the formation of daughter merozoites in segmented schizont stages. Overall, there was no significant difference in the number of merozoites per schizont between PfGSK3β^TGD^ (mean 14.4; range 9–28) and the WT parasite control (mean 14.9; range 8–24) (Fig. 3*D*).

### PfGSK3β is crucial for gametocyte maturation

**Figure 4:**
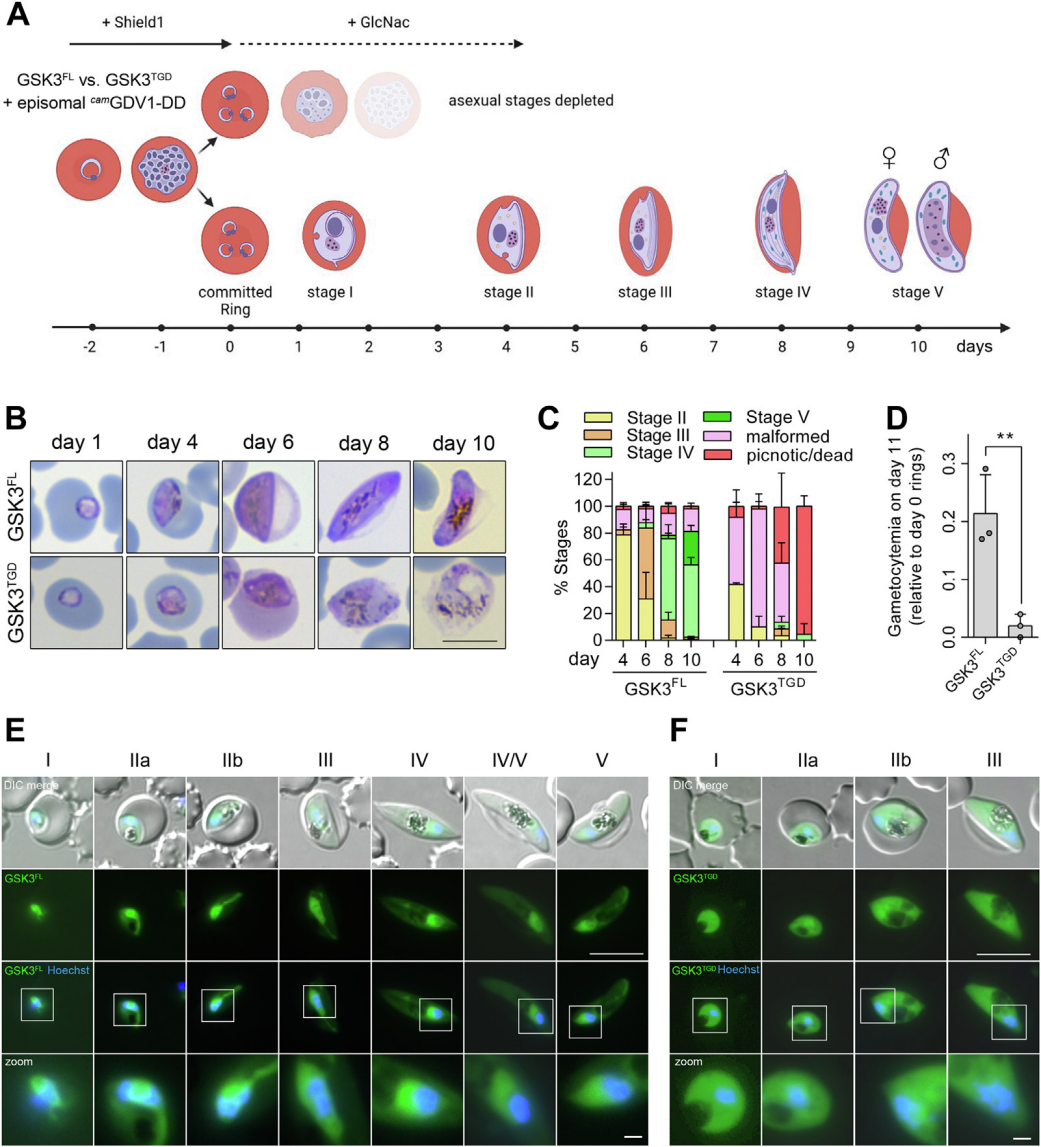
**PfGSK3 is crucial for gametocyte maturation**. *A*, schematic of experimental setup for gametocyte induction. The scheme was created using BioRender (www.biorender.com). *B*, representative images of Giemsa-stained gametocyte stages I to V of PfGSK3β^FL^*versus* PfGSK3β^TGD^. Scale bar = 8 μm. *C*, stage quantification of PfGSK3β^FL^*versus* PfGSK3β^TGD^ on day 4, 6, 8, and 10 of gametocyte development. Percentages represent mean ± SD of three biological replicates. For all time points, a total number of at least 300 parasites were counted. *D*, quantification of gametocytemia on day 11 of gametocyte development for PfGSK3β^FL^*versus* PfGSK3β^TGD^ measured by flow cytometry. Gametocytemia was normalized to ring stage parasitemia on day 0 after induction of sexual commitment. Bar graphs represent mean ± SD of three biological replicates. Significance was analyzed in GraphPad Prism using unpaired two-tailed *t* test (∗*p* < 0.01). *E* and *F*, localization of endogenously GFP-tagged PfGSK3β^FL^ (*E*) and PfGSK3β^TGD^ (*F*) in gametocyte stages analyzed by fluorescence microscopy in unfixed parasites. Nuclei were stained with Hoechst33342. *White squares* refer to magnified sections presented in the bottom panel. DIC, differential interference contrast. Scale bar = 8 μm (zoom 1 μm). GDV1, gametocyte development 1; GSK3, glycogen synthase kinase 3; TGD, targeted gene disruption.

The published expression data (26, 47, 48, 49) also indicate that PfGSK3β is expressed during gametocyte development. To assess the essentiality of PfGSK3β for the development of sexual gametocyte stages, we generated a PfGSK3β-disrupted (and control) cell line allowing conditional induction of sexual commitment. As previously described, sexual commitment of *P. falciparum* can be induced by overexpression of GDV1 (gametocyte development 1) protein providing a robust tool to study gametocyte biology (50, 51). We therefore transfected PfGSK3β^FL^ (Fig. S1*A*) and PfGSK3β^TGD^ (Fig. 1*A*) parasites with an episomal copy of GDV1 fused to GFP and a destabilization domain under the constitutive *cam* promoter and induced sexual commitment by the addition of Shield-1 (Fig. 4*A*) as described recently (52). After induction of sexual commitment, stage conversion as well as localization of PfGSK3β^FL^ *versus* PfGSK3β^TGD^ during gametocyte maturation were analyzed in Giemsa-stained blood smears and by fluorescence microscopy.

First, we analyzed the localization of FL and disrupted PfGSK3β in gametocyte stages using fluorescence microscopy. FL PfGSK3β was detectable in all stages of gametocyte development and localized mainly in the nuclear periphery of all gametocyte stages, reminiscent of its localization in asexual blood stages (Fig. 4*E*). In contrast, the truncated, inactive version expressed in PfGSK3β^TGD^ showed again a solely cytosolic signal (Fig. 4*F*).

Next, we tested the maturation and viability of gametocytes in PfGSK3β^FL^ *versus* PfGSK3β^TGD^. After induction of gametocytogenesis, PfGSK3β^FL^ parasites showed characteristic maturation and stage transition from stage I to stage V within 10 days after sexual commitment (Fig. 4, *B* and *C*). In contrast, gametocytes from PfGSK3β^TGD^ were not able to develop into mature stage V gametocytes (Fig. 4, *B* and *C*). From day 4 onward, PfGSK3β^TGD^ gametocytes developed a strikingly malformed morphology leading to the death of parasites on day 10 (Fig. 4*B*). This death phenotype was quantified by flow cytometry. In agreement with our stage quantification microscopy data, sexually committed PfGSK3β^TGD^ parasites were not detectable on day 11 of gametocyte development (Fig. 4*D*).

Taken together, these findings confirm that PfGSK3β is expressed in sexual gametocyte stages of the parasites and that the kinase is crucial for maturation and survival of gametocytes.

## Discussion

The *P. falciparum* genome encodes for 65 eukaryotic protein kinases (22), of which various members have already been described to be involved in essential processes, including egress, host cell invasion, or gametocytogenesis (17, 19, 38, 53). Nevertheless, to date, only one malaria targeting kinase inhibitor has reached the phase of clinical trials: the PI4K-inhibitor MMV390048 was shown to be safe and well tolerated with high antimalarial activity against all stages of the parasitic life cycle except hypnozoites. Therefore, MMV390048 is considered as a promising partner drug for malaria treatment and prophylaxis (54, 55).

In this study, we aimed to elucidate the role of PfGSK3β in asexual proliferation and gametocytogenesis using reverse genetics to validate this specific enzyme as an antimalarial target. Previous work identified and characterized PfGSK3β inhibitors built on a thieno[2,3-*b*]pyridine or benzofuran scaffold. These inhibitors display potent and selective antimalarial activity with IC_50_ values in the low micromolar to submicromolar range (29, 30, 34). One of these inhibitors has been shown to abrogate RBC invasion by decreasing AMA1 phosphorylation (10). Additionally, some genetic approaches suggested that this parasite kinase is likely to be essential for asexual development (27, 28).

Unexpectedly, using the pSLI vector system (45) we were able to generate PfGSK3β-deficient parasites indicating that this kinase is not essential for survival of asexual blood stages of the parasite *in vitro*. Nevertheless, functional inactivation of PfGSK3β led to a notable defect in parasite multiplication (Fig. 2*A*). Further analysis of this growth phenotype showed that—although PfGSK3β is also expressed in trophozoite stages (23) and shows mainly (peri-)nuclear foci with cytosolic background (10)—the main cellular process that is impaired by PfGSK3β depletion is host cell invasion. This is in line with previous work showing that a hierarchical PfPKA- and PfGSK3β-mediated phosphorylation of the cytoplasmic domain of AMA1 is a prerequisite for efficient RBC invasion (7, 8, 9, 10, 16) and that the use of kinase-specific inhibitors blocks this essential process (8, 10, 36). In contrast to PfPKA, that was shown to be absolutely essential for RBC invasion using conditional knockout systems (9, 16, 19), PfGSK3β does not appear to be as essential for this process (Figs. 2*A* and 3*C*). This partial redundancy of this kinase might be based on (i) that GSK3-specific phosphorylation only enhances the efficiency of RBC invasion or (ii) that another kinase such as PfCK2 can functionally complement PfGSK3β activity. Toward the latter scenario, we analyzed differential expression of the second isoform PfGSK3α without detecting a change in its expression profile; however, upregulation of other kinases cannot be excluded. A similar scenario has already been described for the essential kinase calcium-dependent protein kinase 1 (CDPK1), where inactivation of the latter led to an upregulation of the kinases CDPK5 and CDPK6 (56).

We previously validated the PfGSK3β inhibitors 4h and 4j (30) as potent antimalarial compounds. However, PfGSK3β deficient parasites are viable indicating that the antimalarial activity of the tested inhibitors does not solely rely on inhibition of this enzyme but might target similar ATP binding sites (29, 30) of other kinases. The distinct conservation in ATP-binding pockets of eukaryotic protein kinases is one general obstacle for the design of kinase specific ATP-competing inhibitors (57). Kinases such as PfCK2 with a similar functional profile (6, 17, 18, 58, 59, 60, 61, 62) are conceivable targets.

In a recent study, we provided evidence that autophosphorylation of the activation loop (residues S226 and Y229) is conserved for PfGSK3 and important for folding and solubility of the kinase after recombinant expression in *E.coli* (46). In line with this, we now show that the expression of an inactive mutant (PfGSK3^K96A^) and a phospho-mutant (PfGSK3^S226A/Y229A^) in the parasite result in protein degradation, providing further evidence that autophosphorylation of the activation loop is crucial for folding and solubility of the kinase in its native environment.

In contrast to asexual blood stages, the role and essentiality of protein kinases is much less understood in gametocytes. To date, there are only experimental data on (i) PfCK2 that was shown to be essential for gametocyte maturation and was described as the first essential kinase reported in these stages (17), (ii) PfMAP2 and PfCDPK4 which were reported to be essential in subsequent stages when mature gametocytes form gametes for fertilization (37, 38) and (iii) PfPDK1 that was shown to be involved in gametocyte deformability (19). Conditional knockdown of PfCK2α expression prevented the transition of stage IV into transmission-competent stage V gametocytes, whereas the conditional knockout of PfCK2α completely blocked gametocyte maturation at an earlier stage of sexual differentiation (17). Our study revealed a similar phenotype in PfGSK3β-deficient parasites. However, to date, the specific functions and substrates of PfCK2 and PfGSK3 during gametocytogenesis are not known and need to be addressed in future studies.

The phosphorylation-dependent regulation of essential cellular pathways during gametocyte development is an emerging field and represents potential new drug targets for the experimental development of transmission-blocking antimalarials. Taken together, our study highlights the important role of PfGSK3β in sexual, and to a lesser extent asexual, stages of parasite development, opening up the possibility of targeting this kinase with antimalarials with activity against transmission and disease-causing blood stages.

## Experimental procedures

### Culturing of P. falciparum

Parasites (strain 3D7) (63) were cultured in 0+ erythrocytes from transfusion blood concentrate (Blood bank, Universitätsklinikum Hamburg-Eppendorf) in RPMI complete medium supplemented with 0.5% AlbuMAX at 37 °C in an atmosphere of 1% O_2_, 5% CO_2_, and 94% N_2_ (64). Parasite proliferation was monitored using methanol-fixed, Giemsa-stained blood smears. Parasitemia did not exceed 10%, and the culture media were exchanged at least every second day.

### Cloning of transfection plasmids

The construct pSLI-GSK3-TGD was generated by PCR amplification of 572 bp of the N-terminal GSK3 coding sequence from genomic DNA using primers GSK3_NotI_sense/GSK3_TGD_Mlu_rev (Table S1) and cloning into pSLI-TGD-GFP (45) using NotI/MluI restriction sites.

The construct pSLI-GSK3-GFP was generated by PCR-amplification of 1013 bp of the C-terminal GSK3 coding sequence without a stop codon from genomic DNA using primers GSK3_NotI_sense_SLI/GSK3_AvrII_as_forced_int (Table S1) and cloning into pSLI-GFP-glms (65) using NotI/AvrII restriction sites.

The complementation constructs nmd3_PfGSK3-mCherry were generated by PCR amplification of the GSK3β coding sequence without stop codon. The coding sequence PfGSK3-WT was amplified from *P. falciparum* cDNA using primers GSK3-XhoI_fwd/GSK3_SpeI_rev (Table S1). Mutated sequences (S226A, Y229A, S226A + Y229A) were generated by overlap extension PCR using primers GSK3_S226A_fwd/GSK3_S226A_rev/GSK3_Y229A_fwd/GSK3_Y226A_rev (Table S1) as described previously (46). The coding sequence of PfGSK3-K96A was purchased from GeneScript (46). All coding sequences were inserted into the plasmid nmd3_1xNLS-FRB-mCherry-yDHODH (45) *via* XhoI/SpeI restriction sites thereby replacing the 1xNLS-FRB sequence.

### Generation of transgenic parasite lines

For transfection, mature schizonts were enriched using 60% Percoll and electroporated with 50 μg of plasmid DNA using a Lonza Nucleofector II (66). Transfectants were selected with 4 nM WR99210 (Jacobus Pharmaceuticals) or 0.9 μM DSM1. Stable integrant cell lines were generated as previously described (45). Briefly, parasites carrying the WR99210-selected episomal plasmid were cultivated in the presence of 400 μg/ml G418 (Sigma) to select for integrants with the desired modification of the genomic locus. Correct genomic integration of the construct was confirmed by PCR using genomic DNA from G418-selected integrants and 3D7 WT. Genomic DNA was isolated using QIAamp DNA Mini Kit. For PCR analysis, primers specific for 3′ and 5′ integration as well as WT locus of PfGSK3 were used (Table S1). To generate single clones, parasites were subjected to limiting dilution cloning as described previously (67).

### Imaging

Fluorescence microscopy images of live cells were acquired with a Leica DM6 B microscope equipped with a Leica DFC9000 GT camera using a 100× immersion oil objective. For live cell imaging, parasites were stained with 1 μg/ml DAPI (Biomol) in RPMI for 15 min at 37 °C. Cells were sedimented at 800*g* for 1 min and resuspended in a 1:1 ratio in the supernatant. Ten microliter of the resuspended sample were transferred to a microscopy slide and covered with a cover slip. Images were acquired with Leica Application Suite X (Filter block settings: GFP E×460–500 nm/Em512–542 nm, DAPI E×325–375 nm/Em435–485 nm, mCherry E×542–582 nm/Em604–644 nm) and processed using Adobe Photoshop CS2 with identical settings.

### Immunoblotting

Parasite protein samples of schizont stages (40–44 hpi) were prepared by saponin-lysis of infected erythrocytes and separated on a 12% SDS-PAGE gel at 120 V for 2 h. Separated proteins were transferred to a nitrocellulose membrane (LI-COR) for 1 h at 90 V and 4 °C using a tank blot device (Bio-Rad) according to the manufacturer’s instructions. Membranes were blocked with 5% milk in TBS followed by incubation with primary antibody diluted in 2.5% milk in TBS-T at 4 °C overnight. Primary antibodies were diluted as follows: mouse-anti-GFP (Roche) 1:2000; rabbit-anti-GFP (Thermo Scientific) 1:1000; mouse-anti-RFP (6G6, ChromoTek) 1:2000; and rabbit-anti-BiP (68) 1:2000 in 2.5% milk in TBS-T. After incubation with primary antibodies, membranes were 3× washed with TBS-T and incubated with secondary antibody for 1 h at room temperature in the dark. Secondary antibodies were diluted as follows: goat-anti-mouse800CW (LI-COR) 1:20,000; goat-anti-rabbit800CW (LI-COR) 1:20,000; goat-anti-mouse680RD (LI-COR) 1:20,000; and goat-anti-rabbit680RD (LI-COR) 1:20,000 in 2.5% milk in TBS-T. Membranes were washed again three times with TBS-T and analyzed in an Odyssey FC Imager (LI-COR).

### Flow cytometry

Parasitemia was monitored using flow cytometry as previously described (69). Briefly, 20 μl resuspended parasite culture were transferred to a 1.5 ml reaction tube, and 80 μl RPMI complete medium containing 5 μg/ml dihydroethidium (ThermoFisher) and 1:10,000 SYBR Green (Sigma-Aldrich) were added. Samples were incubated for 20 min at room temperature (protected from light), washed three times with PBS, and analyzed using a NovoCyte 1000 flow cytometer (ACEA Biosciences Inc.). For every sample, 100,0000 events were recorded, and infected RBCs were detected based on SYBR Green fluorescence using NovoExpress software.

### Parasite growth assay

First, late schizont stages were isolated using 60% Percoll purification. Isolated schizonts were taken back into cell culture and incubated at 37 °C for 1 h on a shaker followed by a further incubation for 2 h at standard culture conditions (64) allowing parasites to reinvade new host cells. After reinvasion, parasites were sorbitol-treated to remove unruptured schizonts and obtain a highly synchronous parasite culture with a time window that does not exceed 3 h. Parasites were grown to approx. 40 hpi, and parasitemia was measured by flow cytometry. Afterward, the parasite culture was adjusted to 0.1% parasitemia and 2% hematocrit in 2 ml culture dishes. To monitor parasite proliferation, parasitemia was measured by flow cytometry every 48 h over four parasitic cycles, and culture media were changed before each measurement. After three cycles, the culture was diluted 1:10 to prevent overgrowth, and parasitemia was calculated as cumulative values.

### Quantification of invasion & egress

For phenotypic analysis, schizont parasitemia of a highly synchronous culture was measured by flow cytometry at 40 to 44 hpi as described above. Parasitemia was adjusted to 1%, and schizonts were incubated under static conditions in a 2 ml Petri dish at 5% hematocrit until 52 to 56 hpi to allow for parasite egress and reinvasion to occur. Subsequently, schizont- and ring stage-specific parasitemia were determined in Giemsa-stained blood smears. For this, at least 20 fields of view for each time point were recorded using a 63× objective, and the number of erythrocytes was determined using an automated cell counting software (http://www.gburri.org/parasitemia). To calculate schizont- and ring stage-specific parasitemia, parasite stages were counted manually on recorded images. In total, at least 10,000 cells were analyzed per time point. Egress was quantified as reduction of schizont parasitemia pre-egress (approx. 40–44 hpi) compared to the time after schizont rupture (approx. 52–56 hpi). Invasion was calculated as rings per ruptured schizont using the equation [rings at 56 hpi]/[(schizonts at 44 hpi)-(schizonts at 56 hpi)].

### qPCR

Highly synchronous rings stage parasites were obtained as described above (parasite growth assay) and harvested at 40 hpi. For erythrocyte lysis and storage of RNA, harvested cells were resuspended in prewarmed TRIzol using 10× volume of the sedimented cell pellet. Quantitative real-time PCR was performed in a LightCycler 480 (software version 1.5; Roche). Therefore, RNA purification and Dnase treatment was performed as previously described (70). Prior to use of purified RNA samples, the absence of genomic DNA was confirmed using the LightCycler480. For qPCR analysis, purified RNA was reverse transcribed using Super Script III reverse transcriptase and random hexamer oligonucleotides (Life Technologies). Subsequently, a reaction mix containing 5 μl Quanti Tect SYBR Green PCR Reagent, 0.3 μM forward primer, 0.3 μM reverse primer (Table S1), and 50 ng cDNA was adjusted to a final volume of 10 μl. The reaction mix was incubated at 95 °C for 15 min followed by 40 cycles of elongation for 1 min at 60 °C with subsequent annealing (50–60 °C). The expression of the target gene was normalized against a housekeeping gene (*arginyl-tRNA synthetase*, PF3D7_1218600) as previously described (71, 72) and analyzed using dCt method (73).

### Gametocyte assay

Sexual gametocyte stages were produced by inducible overexpression of the sexual commitment factor GDV1 (*gametocyte development 1*) as previously described (50, 51). Integrant cell lines were transfected with an episomal copy of GDV1-GFP fused to a destabilization domain under the constitutive *cam* promoter (52) allowing induction of GDV1 expression upon addition of the small molecule Shield-1. Sexual commitment was induced by the addition of 4 μM Shield-1 to a culture containing 1% rings. Parasites were cultured for a further 48 h to allow egress and reinvasion. After reinvasion, sexual committed ring stages (day 0) were cultured in RPMI medium supplemented with 0.25% AlbuMAX and 5% human serum (blood group AB+) for 10 days to allow gametocyte maturation. To deplete noncommitted asexual stages, parasites were treated with 50 mM N-acetyl-D-glucosamine. Culture medium was changed daily on a 37 °C heating plate. Stage transition was monitored in Giemsa-stained blood smears, and parasitemia was measured by flow cytometry.

## Data availability

All data are contained within the manuscript. Any additional information required to reanalyze the data reported in this paper is available from the lead contact upon request (gilberger@bnitm.de).

## Supporting information

This article contains supporting information (71).

## Conflict of interest

The authors declare that they have no conflicts of interest with the contents of this article.

## References

[1] WHO (2021).

[2] Bhatt S., Weiss D.J., Cameron E., Bisanzio D., Mappin B., Dalrymple U. (2015). The effect of malaria control on Plasmodium falciparum in Africa between 2000 and 2015. Nature.

[3] Ashley E.A., Dhorda M., Fairhurst R.M., Amaratunga C., Lim P., Suon S. (2014). Spread of artemisinin resistance in Plasmodium falciparum malaria. N. Engl. J. Med..

[4] Burns A.L., Dans M.G., Balbin J.M., de Koning-Ward T.F., Gilson P.R., Beeson J.G. (2019). Targeting malaria parasite invasion of red blood cells as an antimalarial strategy. FEMS Microbiol. Rev..

[5] Adderley J., Williamson T., Doerig C. (2021). Parasite and host erythrocyte kinomics of Plasmodium infection. Trends Parasitol..

[6] Engelberg K., Paul A.S., Prinz B., Kono M., Ching W., Heincke D. (2013). Specific phosphorylation of the PfRh2b invasion ligand of Plasmodium falciparum. Biochem. J..

[7] Treeck M., Zacherl S., Herrmann S., Cabrera A., Kono M., Struck N.S. (2009). Functional analysis of the leading malaria vaccine candidate AMA-1 reveals an essential role for the cytoplasmic domain in the invasion process. PLoS Pathog..

[8] Leykauf K., Treeck M., Gilson P.R., Nebl T., Braulke T., Cowman A.F. (2010). Protein kinase a dependent phosphorylation of apical membrane antigen 1 plays an important role in erythrocyte invasion by the malaria parasite. PLoS Pathog..

[9] Patel A., Perrin A.J., Flynn H.R., Bisson C., Withers-Martinez C., Treeck M. (2019). Cyclic AMP signalling controls key components of malaria parasite host cell invasion machinery. PLoS Biol..

[10] Prinz B., Harvey K.L., Wilcke L., Ruch U., Engelberg K., Biller L. (2016). Hierarchical phosphorylation of apical membrane antigen 1 is required for efficient red blood cell invasion by malaria parasites. Sci. Rep..

[11] Alam M.M., Solyakov L., Bottrill A.R., Flueck C., Siddiqui F.A., Singh S. (2015). Phosphoproteomics reveals malaria parasite Protein Kinase G as a signalling hub regulating egress and invasion. Nat. Commun..

[12] Kumar S., Kumar M., Ekka R., Dvorin J.D., Paul A.S., Madugundu A.K. (2017). PfCDPK1 mediated signaling in erythrocytic stages of Plasmodium falciparum. Nat. Commun..

[13] Bansal A., Singh S., More K.R., Hans D., Nangalia K., Yogavel M. (2013). Characterization of Plasmodium falciparum calcium-dependent protein kinase 1 (PfCDPK1) and its role in microneme secretion during erythrocyte invasion. J. Biol. Chem..

[14] Collins C.R., Hackett F., Strath M., Penzo M., Withers-Martinez C., Baker D.A. (2013). Malaria parasite cGMP-dependent protein kinase regulates blood stage merozoite secretory organelle discharge and egress. PLoS Pathog..

[15] More K.R., Kaur I., Giai Gianetto Q., Invergo B.M., Chaze T., Jain R. (2020). Phosphorylation-dependent assembly of a 14-3-3 mediated signaling complex during red blood cell invasion by Plasmodium falciparum merozoites. mBio.

[16] Wilde M.-L., Triglia T., Marapana D., Thompson J.K., Kouzmitchev A.A., Bullen H.E. (2019). Protein kinase A is essential for invasion of Plasmodium falciparum into human erythrocytes. mBio.

[17] Hitz E., Grüninger O., Passecker A., Wyss M., Scheurer C., Wittlin S. (2021). The catalytic subunit of Plasmodium falciparum casein kinase 2 is essential for gametocytogenesis. Commun. Biol..

[18] Tham W.-H., Lim N.T.Y., Weiss G.E., Lopaticki S., Ansell B.R.E., Bird M. (2015). Plasmodium falciparum adhesins play an essential role in signalling and activation of invasion into human erythrocytes. PLoS Pathog..

[19] Hitz E., Wiedemar N., Passecker A., Graça B.A.S., Scheurer C., Wittlin S. (2021). The 3-phosphoinositide-dependent protein kinase 1 is an essential upstream activator of protein kinase A in malaria parasites. PLoS Biol..

[20] Wadhwa P., Jain P., Jadhav H.R. (2020). Glycogen synthase kinase 3 (GSK3): its role and inhibitors. Curr. Top. Med. Chem..

[21] Wang L., Li J., Di L.-J. (2022). Glycogen synthesis and beyond, a comprehensive review of GSK3 as a key regulator of metabolic pathways and a therapeutic target for treating metabolic diseases. Med. Res. Rev..

[22] Ward P., Equinet L., Packer J., Doerig C. (2004). Protein kinases of the human malaria parasite Plasmodium falciparum: the kinome of a divergent eukaryote. BMC Genomics.

[23] Wichers J.S., Scholz J.A.M., Strauss J., Witt S., Lill A., Ehnold L.-I. (2019). Dissecting the gene expression, localization, membrane topology, and function of the Plasmodium falciparum STEVOR protein family. mBio.

[24] Llinás M., Bozdech Z., Wong E.D., Adai A.T., DeRisi J.L. (2006). Comparative whole genome transcriptome analysis of three Plasmodium falciparum strains. Nucl. Acids Res..

[25] Bozdech Z., Llinás M., Pulliam B.L., Wong E.D., Zhu J., DeRisi J.L. (2003). The transcriptome of the intraerythrocytic developmental cycle of Plasmodium falciparum. PLoS Biol..

[26] López-Barragán M.J., Lemieux J., Quiñones M., Williamson K.C., Molina-Cruz A., Cui K. (2011). Directional gene expression and antisense transcripts in sexual and asexual stages of Plasmodium falciparum. BMC Genomics.

[27] Solyakov L., Halbert J., Alam M.M., Semblat J.-P., Dorin-Semblat D., Reininger L. (2011). Global kinomic and localiza-proteomic analyses of the human malaria parasite Plasmodium falciparum. Nat. Commun..

[28] Zhang M., Wang C., Otto T.D., Oberstaller J., Liao X., Adapa S.R. (2018). Uncovering the essential genes of the human malaria parasite Plasmodium falciparum by saturation mutagenesis. Science.

[29] Fugel W., Oberholzer A.E., Gschloessl B., Dzikowski R., Pressburger N., Preu L. (2013). 3,6-Diamino-4-(2-halophenyl)-2-benzoylthieno[2,3-b]pyridine-5-carbonitriles are selective inhibitors of Plasmodium falciparum glycogen synthase kinase-3. J. Med. Chem..

[30] Masch A., Nasereddin A., Alder A., Bird M.J., Schweda S.I., Preu L. (2019). Structure-activity relationships in a series of antiplasmodial thieno[2,3-b]pyridines. Malar. J..

[31] Balasaheb Aher R., Roy K. (2015). First report on exploring classification and regression based QSAR modelling of Plasmodium falciparum glycogen synthase kinase (PfGSK-3) inhibitors. SAR QSAR Environ. Res..

[32] Lande D.H., Nasereddin A., Alder A., Gilberger T.W., Dzikowski R., Grünefeld J. (2021). Synthesis and antiplasmodial activity of bisindolylcyclobutenediones. Molecules.

[33] Droucheau E., Primot A., Thomas V., Mattei D., Knockaert M., Richardson C. (2004). Plasmodium falciparum glycogen synthase kinase-3: molecular model, expression, intracellular localization and selective inhibitors. Biochim. Biophys. Acta.

[34] Moolman C., van der Sluis R., Beteck R.M., Legoabe L.J. (2021). Exploration of benzofuran-based compounds as potent and selective Plasmodium falciparum glycogen synthase kinase-3 (PfGSK-3) inhibitors. Bioorg. Chem..

[35] Triglia T., Healer J., Caruana S.R., Hodder A.N., Anders R.F., Crabb B.S. (2000). Apical membrane antigen 1 plays a central role in erythrocyte invasion by Plasmodium species. Mol. Microbiol..

[36] Buskes M.J., Harvey K.L., Richards B.J., Kalhor R., Christoff R.M., Gardhi C.K. (2016). Antimalarial activity of novel 4-cyano-3-methylisoquinoline inhibitors against Plasmodium falciparum: design, synthesis and biological evaluation. Org. Biomol. Chem..

[37] Kumar S., Haile M.T., Hoopmann M.R., Tran L.T., Michaels S.A., Morrone S.R. (2021). Plasmodium falciparum calcium-dependent protein kinase 4 is critical for male gametogenesis and transmission to the mosquito vector. mBio.

[38] Hitz E., Balestra A.C., Brochet M., Voss T.S. (2020). PfMAP-2 is essential for male gametogenesis in the malaria parasite Plasmodium falciparum. Sci. Rep..

[39] Josling G.A., Williamson K.C., Llinás M. (2018). Regulation of sexual commitment and gametocytogenesis in malaria parasites. Annu. Rev. Microbiol..

[40] Hawking F., Wilson M.E., Gammage K. (1971). Evidence for cyclic development and short-lived maturity in the gametocytes of Plasmodium falciparum. Trans. R. Soc. Trop. Med. Hyg..

[41] Khan S.M., Franke-Fayard B., Mair G.R., Lasonder E., Janse C.J., Mann M. (2005). Proteome analysis of separated male and female gametocytes reveals novel sex-specific Plasmodium biology. Cell.

[42] Janse C.J., Kroeze H., van Wigcheren A., Mededovic S., Fonager J., Franke-Fayard B. (2011). A genotype and phenotype database of genetically modified malaria-parasites. Trends Parasitol..

[43] Tewari R., Straschil U., Bateman A., Böhme U., Cherevach I., Gong P. (2010). The systematic functional analysis of Plasmodium protein kinases identifies essential regulators of mosquito transmission. Cell Host Microbe.

[44] Gomes A.R., Bushell E., Schwach F., Girling G., Anar B., Quail M.A. (2015). A genome-scale vector resource enables high-throughput reverse genetic screening in a malaria parasite. Cell Host Microbe.

[45] Birnbaum J., Flemming S., Reichard N., Soares A.B., Mesén-Ramírez P., Jonscher E. (2017). A genetic system to study Plasmodium falciparum protein function. Nat. Met..

[46] Pazicky S., Alder A., Mertens H., Svergun D., Gilberger T., Löw C. (2022). N-terminal phosphorylation regulates the activity of glycogen synthase kinase 3 from Plasmodium falciparum. Biochem. J..

[47] Lasonder E., Rijpma S.R., van Schaijk B.C.L., Hoeijmakers W.A.M., Kensche P.R., Gresnigt M.S. (2016). Integrated transcriptomic and proteomic analyses of P. Falciparum gametocytes: Molecular insight into sex-specific processes and translational repression. Nucl. Acids Res..

[48] Pelle K.G., Oh K., Buchholz K., Narasimhan V., Joice R., Milner D.A. (2015). Transcriptional profiling defines dynamics of parasite tissue sequestration during malaria infection. Genome Med..

[49] Young J.A., Fivelman Q.L., Blair P.L., de la Vega P., Le Roch K.G., Zhou Y. (2005). The Plasmodium falciparum sexual development transcriptome: a microarray analysis using ontology-based pattern identification. Mol. Biochem. Parasitol..

[50] Filarsky M., Fraschka S.A., Niederwieser I., Brancucci N.M.B., Carrington E., Carrió E. (2018). GDV1 induces sexual commitment of malaria parasites by antagonizing HP1-dependent gene silencing. Science.

[51] Boltryk S.D., Passecker A., Alder A., Carrington E., van de Vegte-Bolmer R.W., van Gemert G.J. (2021). CRISPR/Cas9-engineered inducible gametocyte producer lines as a valuable tool for *Plasmodium falciparum* malaria transmission research. Nat. Commun..

[52] Wichers J.S., Mesén-Ramírez P., Fuchs G., Yu-Strzelczyk J., Stäcker J., von Thien H. (2022). PMRT1, a plasmodium-specific parasite plasma membrane transporter, is essential for asexual and sexual blood stage development. mBio.

[53] Cassiano G.C., Tavella T.A., Nascimento M.N., Rodrigues D.A., Cravo P.V.L., Andrade C.H. (2021). Targeting malaria protein kinases. Adv. Protein Chem. Struct. Biol..

[54] Sinxadi P., Donini C., Johnstone H., Langdon G., Wiesner L., Allen E. (2020). Safety, tolerability, pharmacokinetics, and antimalarial activity of the novel Plasmodium phosphatidylinositol 4-kinase inhibitor MMV390048 in healthy volunteers. Antimicrob. Agents Chemother..

[55] McCarthy J.S., Donini C., Chalon S., Woodford J., Marquart L., Collins K.A. (2020). A phase 1, placebo-controlled, randomized, single ascending dose study and a volunteer infection study to characterize the safety, pharmacokinetics, and antimalarial activity of the Plasmodium phosphatidylinositol 4-kinase inhibitor MMV390048. Clin. Infect. Dis. Off. Publ. Infect. Dis. Soc. Am..

[56] Bansal A., Ojo K.K., Mu J., Maly D.J., Van Voorhis W.C., Miller L.H. (2016). Reduced activity of mutant calcium-dependent protein kinase 1 is compensated in Plasmodium falciparum through the action of protein kinase G. mBio.

[57] Cohen P. (2002). Protein kinases—the major drug targets of the twenty-first century?. Nat. Rev. Drug Discov..

[58] Dastidar E.G., Dayer G., Holland Z.M., Dorin-Semblat D., Claes A., Chêne A. (2012). Involvement of Plasmodium falciparum protein kinase CK2 in the chromatin assembly pathway. BMC Biol..

[59] Holland Z., Prudent R., Reiser J.-B., Cochet C., Doerig C. (2009). Functional analysis of protein kinase CK2 of the human malaria parasite Plasmodium falciparum. Eukaryot. Cell.

[60] Pease B.N., Huttlin E.L., Jedrychowski M.P., Talevich E., Harmon J., Dillman T. (2013). Global analysis of protein expression and phosphorylation of three stages of Plasmodium falciparum intraerythrocytic development. J. Proteome Res..

[61] Florens L., Washburn M.P., Raine J.D., Anthony R.M., Grainger M., Haynes J.D. (2002). A proteomic view of the Plasmodium falciparum life cycle. Nature.

[62] Le Roch K.G., Zhou Y., Blair P.L., Grainger M., Moch J.K., Haynes J.D. (2003). Discovery of gene function by expression profiling of the malaria parasite life cycle. Science.

[63] Walliker D., Quakyi I.A., Wellems T.E., McCutchan T.F., Szarfman A., London W.T. (1987). Genetic analysis of the human malaria parasite Plasmodium falciparum. Science.

[64] Trager W., Jensen J.B. (1976). Human malaria parasites in continuous culture. Science.

[65] Burda P.-C., Crosskey T., Lauk K., Zurborg A., Söhnchen C., Liffner B. (2020). Structure-based identification and functional characterization of a lipocalin in the malaria parasite Plasmodium falciparum. Cell Rep..

[66] Moon R.W., Hall J., Rangkuti F., Ho Y.S., Almond N., Mitchell G.H. (2013). Adaptation of the genetically tractable malaria pathogen Plasmodium knowlesi to continuous culture in human erythrocytes. Proc. Natl. Acad. Sci. U. S. A..

[67] Thomas J.A., Collins C.R., Das S., Hackett F., Graindorge A., Bell D. (2016). Development and application of a simple plaque assay for the human malaria parasite Plasmodium falciparum. PLoS One.

[68] Struck N.S., de Souza Dias S., Langer C., Marti M., Pearce J.A., Cowman A.F. (2005). Re-defining the Golgi complex in plasmodium falciparum using the novel Golgi marker PfGRASP. J. Cell Sci..

[69] Malleret B., Claser C., Ong A.S.M., Suwanarusk R., Sriprawat K., Howland S.W. (2011). A rapid and robust tri-color flow cytometry assay for monitoring malaria parasite development. Sci. Rep..

[70] Bachmann A., Petter M., Krumkamp R., Esen M., Held J., Scholz J.A.M. (2016). Mosquito passage dramatically changes var gene expression in controlled human Plasmodium falciparum infections. PLoS Pathog..

[71] Wichers J.S., van Gelder C., Fuchs G., Ruge J.M., Pietsch E., Ferreira J.L. (2021). Characterization of apicomplexan amino acid transporters (ApiATs) in the malaria parasite Plasmodium falciparum. mSphere.

[72] Dzikowski R., Frank M., Deitsch K. (2006). Mutually exclusive expression of virulence genes by malaria parasites is regulated independently of antigen production. PLoS Pathog..

[73] Livak K.J., Schmittgen T.D. (2001). Analysis of relative gene expression data using real-time quantitative PCR and the 2(-Delta Delta C(T)) Method. Methods.

