# Supplementary material for: Functional inactivation of Plasmodium falciparum glycogen synthase kinase GSK3 modulates erythrocyte invasion and blocks gametocyte maturation
Source: J Biol Chem. 2022 Aug 10;298(9):102360. doi: 10.1016/j.jbc.2022.102360 (PMC9478393; doi:10.1016/j.jbc.2022.102360)
Supplement: Supporting information [file mmc1.pdf]

Supporting Information

Functional inactivation of Plasmodium falciparum glycogen synthase kinase GSK3 modulates erythrocyte invasion and blocks gametocytogenesis

Arne Alder, Louisa Wilcke, Emma Pietsch, Heidrun von Thien, Samuel Pazicky, Christian Löw, Paolo Mesen-Ramirez, Anna Bachmann, Paul-Christian Burda, Conrad Kunick, Holger Sondermann, Danny Wilson, Tim-Wolf Gilberger

Content:

-Figure S1: Endogenous GFP-tagging of full-length PfGSK3β

-Figure S2: GFP-positive parasites in PfGSK3β^{TGD} (related to Figure 1C)

-Figure S3: Full length Western blot (related to Figure 2E)

-Table S1: Oligonucleotides used in this study

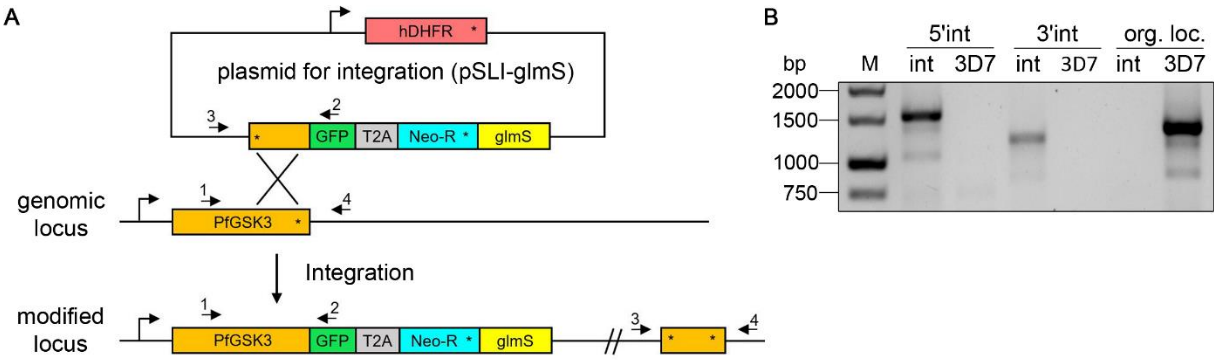

Figure S1: Endogenous GFP-tagging of full-length PfGSK3β. (A) Schematic representation of Selection Linked Integration (SLI) strategy used for endogenous GFP-tagging of PfGSK3β. Binding sites of primers used to confirm correct genomic integration are indicated by black arrows. SLI = Selection Linked Integration; hDHFR = human dihydrofolate reductase; GFP = green fluorescent protein; T2A = skip peptide; Neo-R = neomycin-resistance gene; glmS = glmS ribozyme in 3'UTR; angled arrows = promoters; asterisks = stop codon. **(B)** Integration PCR with genomic DNA isolated from integrant parasites (int) or 3D7 wild-type parasites (3D7). Primer combinations: 5'int (1+2); 3'int (3+4); wild-type locus (org. loc.) (1+4). Position of primers as indicated in (A). Expected PCR products: 1+2 = 1556 bp; 3+4 = 1234 bp; 1+4 = 1344 bp.

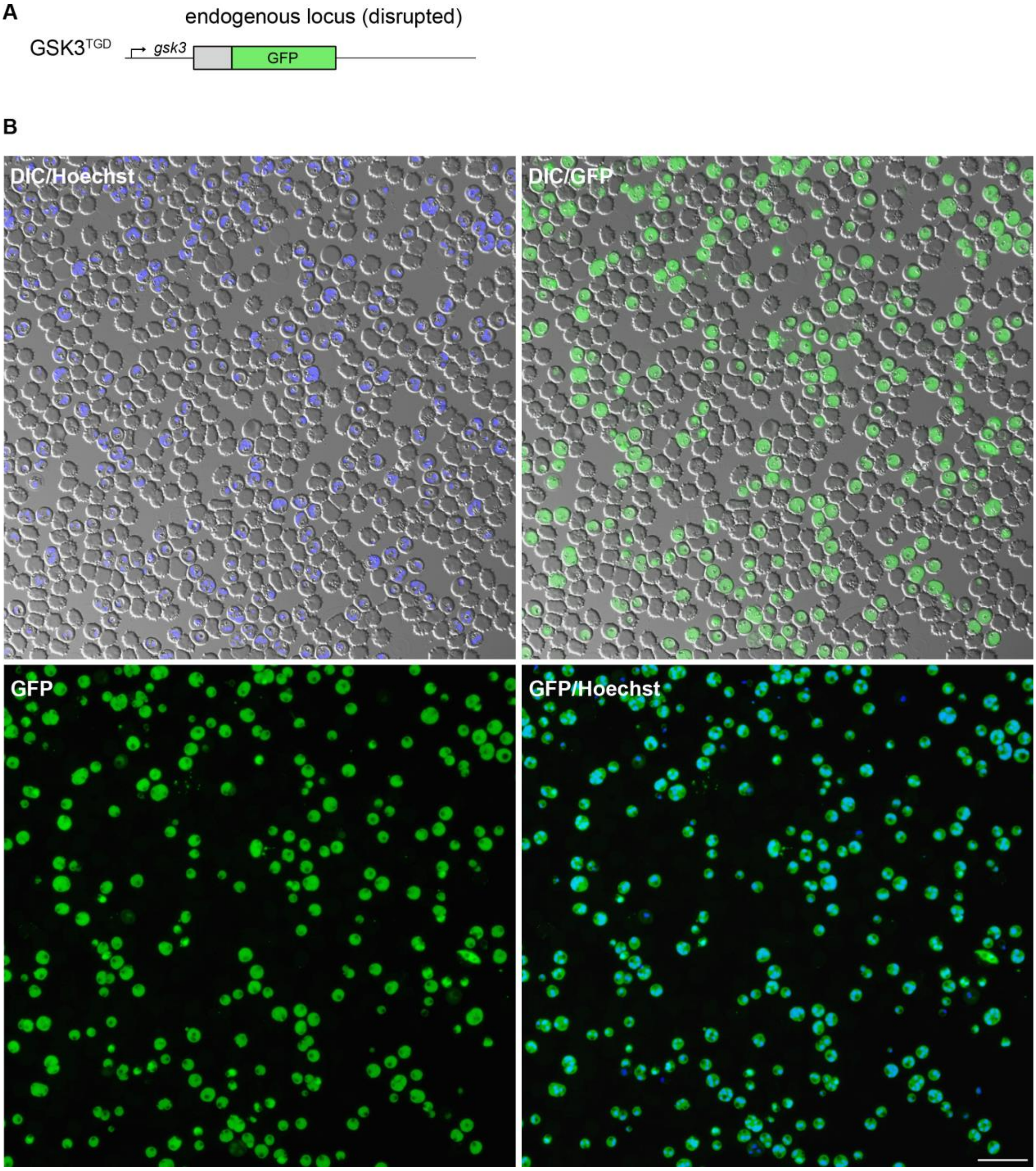

Figure S2: GFP-positive parasites in PfGSK3β^{TGD}, related to Figure 1C. (A) Schematic representation of truncated PfGSK3β as GFP fusion after disruption of the genomic locus by SLI-TGD. **(B)** Representative fluorescence microscopy of GFP-expressing PfGSK3β^{TGD} parasites. Nuclei were stained with Hoechst33342. DIC = Differential interference contrast. Scale bar = 20 µm.

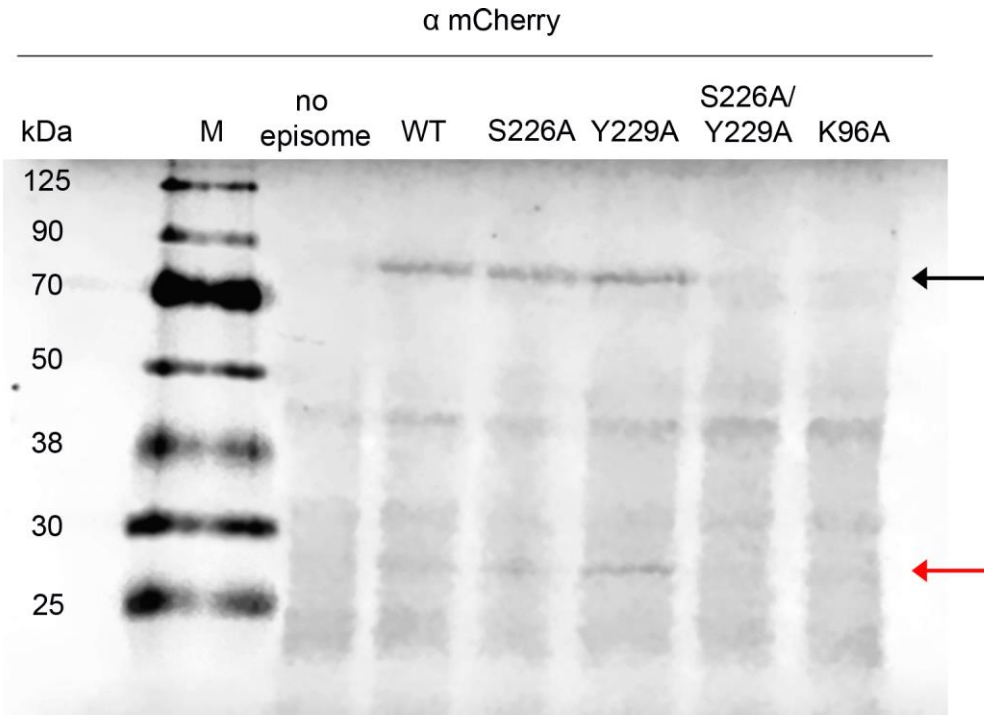

Figure S3: Full length Western blot, related to Figure 2E: Episomally expressed versions of PfGSK3β-mCherry (~80 kDa, point mutations as indicated) were detected using anti-RFP antibodies. PfGSK3β^{TGD} without episomal complementation (no episome) was used as a negative control. Black arrow indicates band corresponding to MW of PfGSK3β-mCherry (80 kDa); red arrow indicates band corresponding to MW of mCherry (26 kDa).

Table S1: Oligonucleotides used in this study

Oligo name	Oligo sequence[a]	Purpose
GSK3_NotI_sense_SLI	gcgcgcggccgcTAACGGGACAGTCAAGTGTGG	Cloning PfGSK3β^{FL} + PfGSK3β^{TGD}
GSK3_AvrII_as_forced_int	gcgccctaggACTTTCTATGATAACGTGCG	
GSK3_TGD_NotI_TAA_fwd	GCGCgcggccgcTAAAAAAATTGGCCTATAGGTATAGAG	
GSK3_TGD_MluI_rev	gcgcacgcgtAACACCAAAACTACCATTCCCTAT	
GSK3_TGD_inte check_rev	ATAGATACGTCAGAACAAGTTGCGA	integration check PCR PfGSK3β^{TGD} + PfGSK3β^{FL}
PfGSK3_5'UTR_125_fwd	CCTTTTGTCAATTTTGTAATTATAC	
GSK3_inte check 1221bp_sense	GCGCGCTCAACAAATTACACAACAC	
GSK3_3'UTR_120_rev	GCGCATGTTTACAGTTTAATTATTTCCTG	
GFP272_as	CCTTCGGGCATGGCACT	
pARL55_sense	GGAATTGTGAGCGGATAACAATTTCACACAGG	
GSK3B_XhoI_fwd	gcgcctcgagATGAAAAATTGGCCTATAGA	Cloning episomal PfGSK3β WT + mutants
GSK3B_SpeI_rev	gcgcactagtACTTTCTATGATAACGTGCG	
GSK3B_S226A_fwd	TCTATTAGCTGGGCAAAGAGCTGTCTCATATATTTGTTCAAGGTTTTATCG	
GSK3B_S226A_rev	CGATAAAACCTTGAACAAATATATGAGACAGCTCTTTGCCCAGCTAATAGA	
GSK3B_Y229A_fwd	CTGGGCAAAGAAGTGTCTCAGCAATTTGTTCAAGGTTTTATCG	
GSK3B_Y229A_rev	CGATAAAACCTTGAACAAATTGCTGAGACACTTCTTTGCCCAG	
GSK3-I-TGD_qPCR_fwd	AGTGAGGATGAAGATGAGGAAAGA	qPCR *pfgsk3β*
GSK3-I-TGD_qPCR_rev	AACACCAAAACTACCATTCCCT	
MAL13P1.84_WT_qPCR_fwd	ACGTTGGTATAGAGCACCAGA	qPCR *pfgsk3α*
MAL13P1.84_WT_qPCR_rev	TGGAGATCTTTTAATTGCAACTGCT	
arginyl-tRNA synthetase_for (1)	TTCAAAACACGAAGTGGAACAAC	qPCR *arginyl-tRNA synthetase*
arginyl-tRNA synthetase_rev (1)	AATTCTCTGCAGCAAGTCGC	

[a]Annealing bases are shown in upper case letters. Overhangs are denoted with lower case letters. Letters with underline indicate restriction sites.

REFERENCES

1. Wichers, J. S., van Gelder, C., Fuchs, G., Ruge, J. M., Pietsch, E., Ferreira, J. L., Safavi, S., von Thien, H., Burda, P.-C., Mesén-Ramirez, P., Spielmann, T., Strauss, J., Gilberger, T.-W., and Bachmann, A. (2021) Characterization of Apicomplexan Amino Acid Transporters (ApiATs) in the Malaria Parasite Plasmodium falciparum. *mSphere*. **6**, e0074321–e0074321
